# Dasatinib Inhibits Basal B Breast Cancer Through ETS1-Mediated Extracellular Matrix Remodeling

**DOI:** 10.3390/biomedicines13122888

**Published:** 2025-11-26

**Authors:** Xinyu Guo, Heng Sun, Feng Yu, Yangyang Feng, Sen Guo, Josh Haipeng Lei, Kai Miao, Ka-U Ip, Ling Li, Hanghang Li, Xiaohong Liao, Xiaoling Xu, Rong Zhou, Chu-Xia Deng

**Affiliations:** 1 Cancer Centre, Faculty of Health Sciences, University of Macau, Macau SAR, China; yc17668@connect.um.edu.mo (X.G.); hengsun@um.edu.mo (H.S.);; 2 Centre for Precision Medicine Research and Training, Faculty of Health Sciences, University of Macau, Macau SAR, China; 3 MOE Frontier Science Centre for Precision Oncology, University of Macau, Macau SAR, China; 4 State Key Laboratory of Quality Research in Chinese Medicine, Institute of Chinese Medical Sciences, University of Macau, Macau SAR, China; 5 Guangzhou National Laboratory, No. 9 XingDaoHuanBei Road, Guangzhou International Bio Island, Guangzhou 510005, China; 6 State Key Laboratory of Respiratory Disease, National Clinical Research Center for Respiratory Disease, Guangzhou Institute of Respiratory Health, The First Affiliated Hospital of Guangzhou Medical University, Guangzhou Medical University, Guangzhou 510120, China

**Keywords:** triple-negative breast cancer (TNBC), metastasis, dasatinib, extracellular matrix, ETS proto-oncogene 1 (*ETS1*), matrix metalloproteinase-3 (*MMP3*)

## Abstract

**Background/Objectives**: Developing effective therapies for patients with triple-negative breast cancer (TNBC) remains an urgent clinical priority. Compared with other subtypes, the basal B type of TNBC exhibits a less differentiated and mesenchymal-like phenotype that models highly invasive and metastatic breast malignancies. To target metastatic TNBC, our current study sought to identify effective therapeutic drugs and the underlying mechanisms. **Methods**: A systematic screening of 140 FDA-approved drugs was conducted for repurposing using live-cell imaging-based wound-healing assays. Candidate efficacy was validated by in vitro transwell invasion assays, in vivo allograft/xenograft models, and ex vivo three-dimensional air–liquid interface (ALI) and patient-derived organoid (PDO) cultures. **Results**: Dasatinib emerged as a promising anti-cancer agent in aggressive TNBC, particularly in the basal B type, with high ETS proto-oncogene 1 (*ETS1*) expression. Mechanistically, dasatinib disrupts the actin cytoskeleton, impairing cell motility and migration while concurrently suppressing the expression of *ETS1* and matrix metalloproteinase-3 (*MMP3*) to remodel the extracellular matrix (ECM) and inhibit invasion. Moreover, the combination of dasatinib with an anti-programmed cell death protein-1 (PD-1) antibody represents a potential therapeutic strategy. **Conclusions**: These findings highlight dasatinib as a potential therapeutic option for metastatic TNBC and suggest that selecting patients with high ETS1 expression may optimize treatment response.

## 1. Introduction

Breast cancer is the most prevalent malignancy and the leading cause of death among women worldwide [1]. It exhibits substantial heterogeneity, comprising intrinsic molecular subtypes such as luminal A/B, human epidermal growth factor receptor 2 (HER2)-enriched, basal-like, normal-like, and claudin-low types [2,3]. Clinically, breast cancer is categorized by estrogen receptor (ER), progesterone receptor (PR), and HER2 expression into hormone receptor (HR)-positive (~75%), HER2-positive (~10%), and triple-negative breast cancer (TNBC, 10–15%) [2]. Although basal-like tumors have been recognized as TNBC over time, these terms are not synonymous [4]. TNBC/basal-like tumors have been further subclassified into basal A and basal B [5,6]. Compared to basal A (more basal-like, KRT5^+^/KRT14^+^), basal B cancers are less differentiated, exhibit mesenchymal traits (Vim^+^), and display higher stemness markers (CD44^+^/CD24^−^), as well as aggressive invasiveness [5,6], closely resembling claudin-low or metaplastic breast cancers in clinical settings.

Cancer metastasis is orchestrated by dynamic interactions between tumor cells and the microenvironment, particularly via cytoskeletal reorganization and extracellular matrix (ECM) remodeling [7]. During invasion, cancer cells undergo epithelial–mesenchymal transition (EMT), marked by actin cytoskeleton rearrangement via RhoA signaling, which enhances contractility and the formation of membrane protrusions (such as filopodia and invadopodia) [8,9]. Simultaneously, these protrusions facilitate ECM proteolysis through the secretion of metalloproteinases (MMPs), including MMP2 and MMP9, enabling tumor cells to breach basement membranes and intravasate into circulation [10]. ECM remodeling is further reinforced by collagen I and fibronectin through integrin–FAK–SRC signaling, contributing to migration, survival, and drug resistance [11]. The SRC family kinases (SFKs), comprising 11 members [12], represent a key class of non-receptor tyrosine kinases (nRTKs) that regulate cancer progression [13]. SFKs also interact with EGFR and HER2, activating downstream effectors such as STAT3, MAPK, and PI3K–AKT pathways [14,15].

Dasatinib, a potent SFK inhibitor approved by the U.S. FDA for leukemia in 2006, has shown therapeutic potential in breast cancer. A previous study reported that combined treatment with dasatinib and the Myeloid cell leukemia-1 (MCL1) inhibitor (BIMs2A) can suppress breast cancer metastasis [16]. Preclinical studies demonstrate that dasatinib blocks paclitaxel-induced SFK activation, reduces breast cancer stem cells (BCSCs), and enhances chemosensitivity [17]. Clinically, its combination with paclitaxel in HER2-negative metastatic breast cancer achieved a 23% response rate and 5.2 months of median progression-free survival, supporting its role in refractory disease management [18].

Our previous study showed that low-dose cisplatin inhibits breast cancer metastasis via cytoskeletal remodeling with minimal side effects [19]. Based on these results, we hypothesized that other pharmaceutical agents might display similar activities. Hence, we conducted a systematic screening of our in-house library of 140 compounds to identify potential candidates for drug repurposing in the treatment of breast cancer metastasis. Our research identified dasatinib as a promising drug against metastasis. It functions by disrupting the actin cytoskeleton and impeding filopodia formation, thereby impairing cell motility and migration. Additionally, dasatinib downregulates the ETS proto-oncogene 1 (*ETS1*) and matrix metalloproteinase 3 (*MMP3*), leading to ECM remodeling and inhibition of metastasis. This work addresses a critical paradox in dasatinib translation, where clinical trials did not demonstrate benefits in unselected breast cancer patients despite robust preclinical efficacy. By selecting TNBC patients with high ETS1 expression, the therapeutic response to dasatinib may be enhanced, potentially transforming it into a precision oncology tool for aggressive TNBC. Furthermore, combining dasatinib with anti-programmed cell death protein-1 (PD-1) antibodies may offer a potent strategy for metastatic breast cancer treatment.

## 2. Materials and Methods

### 2.1. Cell Culture

All cell lines were obtained from the American Type Culture Collection (ATCC). Cells were routinely cultured in Dulbecco’s Modified Eagle Medium (DMEM; Gibco, Waltham, MA, USA) or Roswell Park Memorial Institute (RPMI) 1640 medium (Gibco), supplemented with 10% fetal bovine serum (FBS; Gibco) and 100 U/mL penicillin–streptomycin (Gibco). Notably, Hs578T cells were maintained in medium additionally supplemented with 10 μg/mL insulin (Sigma, I9278, Burlington, MA, USA). SUM149PT cells were cultured in the DMEM/Nutrient Mixture F-12 (DMEM/F12; Gibco) medium with 10% FBS, 100 U/mL penicillin–streptomycin, 1 μg/mL hydrocortisone, and 10 μg/mL insulin. All cells were incubated in a humidified incubator at 37 °C with 5% carbon dioxide (CO_2_).

### 2.2. Cell Viability Assay

Cells were seeded into 384-well or 96-well plates and incubated overnight to allow attachment. The medium was then replaced with fresh medium containing a range of drug concentrations. The information on 140 drugs was listed in Supplementary Table S1. The drugs were obtained and prepared as 10 mM stock solutions in dimethyl sulfoxide (DMSO) or phosphate-buffered saline (PBS), based on their solubility and molecular weight. After 48 h of treatment, cells were incubated with Alamar Blue (Sigma, R7017) reagent for 2 h at 37 °C. Fluorescence was measured using a SpectraMax M5e Multi-Mode Microplate Reader (Molecular Devices, San Jose, CA, USA) with excitation at 560 nm and emission at 590 nm. Dose–response curves were generated from triplicate experiments, and the half-maximal and 20% inhibitory concentration values (IC_50_ and IC_20_) were calculated using GraphPad Prism 10.2.2 software.

### 2.3. Incucyte 96-Well Scratch Wound-Healing Assays

4T1 cells were seeded at a density of 5 × 10^5^ cells/mL in 96-well plates and cultured to confluence in a humidified incubator at 37 °C with 5% CO_2_ for 24 h. Monolayers were scratched using the 96-pin WoundMaker™ (Essen BioScience, Ann Arbor, MI, USA) to create uniform wounds, followed by two washes with phosphate-buffered saline (PBS) to remove dislodged cells. Subsequently, the medium was replaced with 2% FBS medium containing either vehicle (negative control), 400 nM cisplatin (positive control), or the IC20 concentration of candidate drugs. Wound closure was monitored via time-lapse imaging every 6 h for 36 h using the Incucyte HD Live-Cell Analysis System (Essen BioScience). Relative wound density was quantified using the integrated software, with data representing mean ± SD from triplicate experiments.

### 2.4. Transwell Assay

The upper chamber of the Transwell insert (Corning, 3422, Corning, NY, USA) was coated with Matrigel (Corning, 354234) at a final concentration of 1–2 mg/mL. After the Matrigel solidified, cells were trypsinized and resuspended in serum-free medium at a density of 3 × 10^4^ cells per well and then added to the upper chamber. The lower chamber was filled with medium containing 10% FBS to serve as a chemoattractant. After incubation at 37 °C with 5% CO_2_ for 24 h, non-migrated cells were removed from the upper surface of the membrane using a cotton swab. The migrated cells on the lower surface were fixed with 4% paraformaldehyde for 15 min and stained with a crystal violet solution (Sigma-Aldrich, V5265, Burlington, MA, USA) for 15 min. Images were captured using a light microscope in bright-field mode.

### 2.5. Colony Formation Assay

Cells were plated at a density of 100 cells per well in 6-well plates and cultured for 2 weeks. The culture medium was refreshed every 5 days. After incubation with different doses of dasatinib (Selleckchem, S1021, Houston, TX, USA) for 48 h, the cells were fixed with 95% methanol for 15 min and then stained with crystal violet solution (Sigma-Aldrich, V5265) for 15 min. After washing with water, colonies were counted manually.

### 2.6. Plasmids and Lentivirus Infection

Stable overexpression plasmids, including pLV3-CMV-Mmp3 (mouse)-3×HA-CopGFP-Puro (P73055), pLV3-CMV-MMP3 (human)-3×HA-CopGFP-Puro (P60013), pLV3-CMV-Ets1 (mouse)-3×HA-CopGFP-Puro (P73142), and pLV3-CMV-ETS1 (human)-3×HA-CopGFP-Puro (P73054), were obtained from MiaoLingPlasmid (Wuhan, China). The clustered regularly interspaced short palindromic repeats (CRISPR)-Cas9 lentiCRISPRv2 plasmid (Addgene, #52961, Watertown, MA, USA) was used for a stable knockout system, and single guide RNA (sgRNA) sequences were cloned into the plasmid following standard protocols [20,21]. The sgRNAs used for single-gene knockout were listed in Table 1.

HEK293T cells, cultured in DMEM medium supplemented with 10% FBS, were used to produce lentiviruses. Transfection was performed in 10 cm dishes using polyethyleneimine (PEI; Polyscience, 24765, Warrington, PA, USA) with a mixture of 4.8 μg of target plasmid, 3.6 μg of the viral packaging plasmid pCMV-delta 8.2 (Addgene #12263), and 2 μg of the envelope plasmid pMD2.G vesicular stomatitis virus glycoprotein (VSV-G) (Addgene #12259).

After 48 h, viral supernatants were harvested, filtered through 0.45 μm polyethersulfone (PES) filters, and either used immediately for infection or stored at −80 °C. To establish CRISPR knockout or stable overexpression cell lines, cells were infected with the lentiviruses at a 1:1 dilution in culture medium. Forty-eight hours post-infection, the medium was replaced with fresh medium containing 2 μg/mL puromycin (InvivoGen, ant-pr-1, San Diego, CA, USA) for selection.

### 2.7. Western Blot

Protein lysates were extracted using radioimmunoprecipitation assay (RIPA) buffer (Beyotime, P0013B, Shanghai, China), and protein concentrations were determined using Pierce™ bicinchoninic acid (BCA) Protein Assay Kit (Thermo Fisher Scientific, 23227, Waltham, MA, USA). Samples were denatured using 5× loading buffer solution (250 mM Tris-HCl, pH 6.8; 30% [*v*/*v*] glycerol; 10% sodium dodecyl sulfate [SDS]; 0.05% [*w*/*v*] bromophenol blue; and 10 mM dithiothreitol with 25% 2-mercaptoethanol) and heated at 95 °C for 10 min.

Equal amounts of protein (20 μg per well) were subjected to SDS-polyacrylamide gel electrophoresis (SDS-PAGE) using 10% or 15% gels and transferred to nitrocellulose membranes (Millipore, Burlington, MA, USA). Membranes were blocked with 3% bovine serum albumin (BSA) in tris-buffered saline with Tween 20 (TBS-T; 20 mM Tris; 150 mM NaCl; and 0.1% Tween 20, pH 7.4–7.6) for 1 h at room temperature and then incubated overnight at 4 °C with primary antibodies.

The primary antibodies used were anti-ETS1 (1:1000, cell signaling technology [CST], 14069S, Danvers, MA, USA), anti-MMP3 (1:250, Abcam, ab52915, Burlingame, CA, USA), anti-Caveolin-1 (1:1000, CST, 3238S), anti-actinin-4 (1:1000, Invitrogen MA5-32794, Waltham, MA, USA), anti-paxillin (1:500, Invitrogen, AHO0492), anti-β-actin (1:1000, CST, 3700S), anti-glyceraldehyde 3-phosphate dehydrogenase (GAPDH; 1:1000, Abcam, ab8245), anti-Snail (1:1000, CST, 3879), anti-Vimentin (1:1000, CST, 5741), anti-Vinculin (1:1000, CST, 13901S), and anti-phospho-p44/42 MAPK (pERK1/2; 1:1000, CST, 4370L).

After three washes with TBS-T buffer, membranes were incubated with horseradish peroxidase (HRP)-conjugated secondary antibodies (anti-rabbit IgG, 1:5000, CST, 7074S; anti-mouse IgG, 1:5000, CST, 7076S) for 1 h at room temperature. The membranes were washed three times with TBS-T, and protein bands were visualized using an enhanced chemiluminescence reagent (Millipore, WBULS0500) and detected with a ChemiDoc Imaging System (Bio-Rad, Hercules, CA, USA). Protein levels were quantified using ImageJ 1.54f (Bethesda, MD, USA) and normalized to the reference control.

### 2.8. Immunofluorescence Staining

After three washes with PBS, cells were fixed with 4% paraformaldehyde for 20 min, followed by permeabilization with 0.05% Triton X-100 for 15 min and blocking with 5% BSA for 30 min. Sections were then incubated overnight at 4 °C with primary antibodies diluted in blocking buffer. The dyes and primary antibodies used were PKH26 (1:200, Sigma-Aldrich, PKH 26GL), anti-Paxillin (2 μg/mL, Invitrogen, AHO0492), and Phalloidin (1×, Invitrogen, R415). After three washes with PBS, sections were incubated with Alexa Fluor 488 and Alexa Fluor 647 secondary antibodies (Thermo Fisher Scientific, A-11008, A-11001, A-21245, and A-21235, Waltham, MA, USA) diluted 1:500 in blocking solution at room temperature for 1 h. Subsequently, sections were stained with Hoechst 33342 (1:2000, Invitrogen, H3570) to visualize nuclei. Samples were imaged (63× oil objective) using a Zeiss LSM 880 confocal microscope (Zeiss, Jena, Germany).

### 2.9. Quantitative Real-Time PCR

Total RNA was extracted from the collected samples using TRIzol reagent (Thermo Fisher Scientific, 15596026). Reverse transcription was carried out using a PrimeScript™ reverse transcription (RT) reagent kit (Takara, RR037A, Takarazuka, Japan). Quantitative real-time polymerase chain reaction (qRT-PCR) was performed using FastStart SYBR Green Master Mix (Roche, 04913850001, South San Francisco, CA, USA) and quantified on a QuantStudio™ 7 Flex Real-Time PCR System (Thermo Fisher Scientific).

Relative gene expression levels were normalized to the endogenous reference genes of 18S ribosomal RNA (*18S*) for human samples and β-Actin (*Actb*) for mouse samples. The qRT-PCR primers were listed in Table 2.

### 2.10. Animal Experiments

All animal experiments were conducted in compliance with protocols (UMARE-015-2019) approved by the University of Macau’s Animal Care Ethics Committee and in accordance with Macau’s Council on Animal Care guidelines. Female BALB/c mice or nude mice aged 6–8 weeks were randomly assigned to experimental groups.

For the orthotopic tumor model, mice were anesthetized via intraperitoneal injection of 12.5 mg/mL avertin (Sigma-Aldrich, T48402) containing tert-butyl alcohol (Sigma-Aldrich, 240486) at a dose of 300 μL per mouse. 5 × 10^5^ 4T1 murine mammary carcinoma cells were injected into the mammary fat pad of BALB/c mice. When tumors became visible (day 7), mice were administered dasatinib or vehicle by oral gavage at a dose of 10 mg/kg/day. The vehicle solution was composed of 30% PEG300, 61% double-distilled water, 4% DMSO, and 5% Tween 80. Mice were sacrificed on day 32.

For the metastasis model, 2 × 10^5^ 4T1 cells were injected via the tail vein. Mice received oral gavage of vehicle or dasatinib (10 mg/kg/day). Throughout the experiment, body weight and tumor volume (calculated as V = length × tumor width^2^/2) were measured every two days. Mice were sacrificed on day 14.

### 2.11. Three-Dimensional Ex Vivo Air–Liquid Interface (ALI) Culture

Orthotopic tumors were generated by injecting 5 × 10^5^ 4T1 murine mammary carcinoma cells into the inguinal mammary fat pad of female BALB/c or nude mice; solid masses reaching 5–8 mm in diameter were surgically excised 7–10 days post-inoculation and processed for downstream assays.

Collagen gel matrices for the inserts were prepared by mixing the collagen matrix, 10× concentrated sterile culture medium (Ham’s F-12), and sterile reconstitution buffer (2.2 g sodium bicarbonate [NaHCO_3_] in 100 mL of 0.05 N sodium hydroxide [NaOH] and 200 mM 4-(2-hydroxyethyl)-1-piperazineethanesulfonic acid [HEPES]) on ice at a ratio of 8:1:1 immediately before use [22]. The inserts were then polymerized for 20 min in a 37 °C incubator. Tumor tissue was harvested from mice, excluding necrotic regions, and finely minced on a 500 μM wire mesh before being resuspended in the preprepared collagen mixture. Once the collagen mixture in the inserts had solidified, 50 μL of the tumor cell suspension was carefully added to each insert, avoiding bubble formation.

After an additional 30 min incubation to allow solidification, culture medium was gently added to the bottom layer to establish the air–liquid interface culture. The medium was replaced on day 4, and cell viability was assessed on day 7 using the 3-(4,5-dimethylthiazol-2-yl)-2,5-diphenyltetrazolium bromide (MTT) assay. After imaging, the ALI tissue was dissolved in dimethyl sulfoxide (DMSO) for the quantification of cell viability.

### 2.12. Public Data Collection and Processing

The IC_50_ values for treatment with various compounds and the bulk RNA sequencing (RNA-seq) data from diverse human cancer cell lines were obtained from the Genomics of Drug Sensitivity in Cancer 2 (GDSC2) database https://www.cancerrxgene.org/ [23] and the Cancer Dependency Map (DepMap) database (Version: V24Q4) https://depmap.org/portal/ [24] (accessed on 16 January 2025). RNA-seq and clinical data from breast invasive carcinoma (BRCA) samples were obtained from The Cancer Genome Atlas (TCGA) database https://portal.gdc.cancer.gov/ (accessed on 2 September 2024).

### 2.13. Sample Preparation and RNA-Seq Process

Total RNA was extracted from cultured cells using TRIzol reagent (Thermo Fisher Scientific, 15596026, Waltham, MA, USA) and then purified using the RNeasy mini kit (QIAGEN, 74104, Germantown, MD, USA). The RNA concentration and integrity were assessed using the Agilent 2100 Bioanalyzer (Agilent Technologies, Santa Clara, CA, USA), with RNA integrity number (RIN) value exceeding 7.0. RNA quantity was further verified with the Qubit 2.0 Fluorometer (Invitrogen). Complementary DNA (cDNA) libraries for RNA-seq were prepared using the NEBNext^®^ Ultra™ RNA Library Prep Kit for Illumina (New England Biolabs, E7770, Ipswich, MA, USA) and subjected to quality control. Sequencing was conducted on the NovaSeq 6000 platform (Illumina, San Diego, CA, USA) using 150-base pair paired-end reads.

### 2.14. RNA-Seq Data Preprocessing and Analysis

Raw reads from sequencing results were quality-checked using FastQC v0.12.1 software [25], and summary results were compiled using MultiQC v1.16 [26]. Adapter sequences were trimmed using Trim Galore to generate clean reads. Sequence alignment was performed using Spliced Transcripts Alignment to a Reference (STAR, v2.5.2b) [27] against the human genome (hg38) or mouse genome (mm19) using default parameters. Gene-level read quantification was performed using FeatureCounts (v1.6.0) [28]. Principal component analysis (PCA) was used to evaluate batch effects in gene expression across different experimental conditions. Differentially expressed gene (DEG) analysis was carried out using DESeq2 [29]. Genes with adjusted *p*-value < 0.05 were considered statistically significant and were selected for downstream functional enrichment analysis, including Gene Ontology (GO) and Kyoto Encyclopedia of Genes and Genomes (KEGG) pathway analysis using the R package “clusterProfiler” 4.14.4. All *p*-values were adjusted according to the Benjamini–Hochberg method for multiple testing correction. Volcano plots, heatmaps, and bar plots were generated using R software (version 4.2.0).

### 2.15. Statistical Analysis

All values on the graphs are presented as mean ± standard deviation (SD) from triplicate experiments. Statistical analysis was performed using GraphPad Prism Version 10.0 (GraphPad Software, San Diego, CA, USA). For comparisons between two groups, a two-sided Student’s T-test was used. One-way analysis of variance was used for comparisons among multiple groups. Differences were considered statistically significant at *p* < 0.05. Correlation analysis was performed using the Pearson correlation coefficient or the nonparametric Spearman correlation. The levels of statistical significance are indicated as follows: * *p* < 0.05, ** *p* < 0.01, *** *p* < 0.001, and **** *p* < 0.0001.

## 3. Results

### 3.1. Large-Scale Drug Screening Identifies Dasatinib as an Inhibitor for Breast Cancer Cell Migration

**Figure 1:**
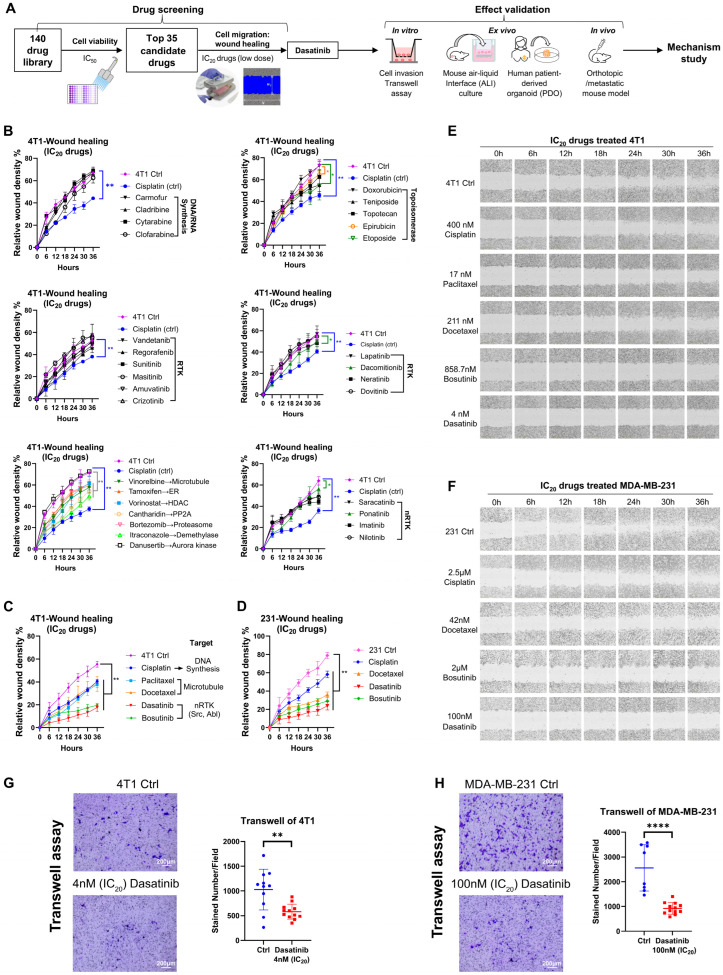
Screening of 140 drugs for inhibition of breast cancer cell migration. (**A**) Schematic overview of the drug screening workflow. (**B**–**D**) Assessment of anti-migratory effects of the top 35 drugs. Wound-healing assays were performed at IC_20_ concentrations of the drugs, with images captured every 6 h over a 36 h period using the Incucyte live-cell imaging system. Migration was quantified by relative wound density. Error bars represent the mean ± SD from triplicate experiments. Abbreviations: RTK, receptor tyrosine kinase; ER, estrogen receptor; HDAC, histone deacetylase; PP2A, protein phosphatase 2. (**E**) Representative wound-healing images of 4T1 cells. (**F**) Representative wound-healing images of MDA-MB-231 cells. (**G**) Transwell invasion assay of 4T1 cells treated with dasatinib at the IC_20_ concentration. Representative images and quantification from triplicate experiments are shown. (**H**) Transwell invasion assay of MDA-MB-231 cells treated with dasatinib at the IC_20_ concentration. Representative images and quantification from triplicate experiments are shown. Statistical analysis was determined using a two-tailed *t*-test. * *p* < 0.05, ** *p* < 0.01, **** *p* < 0.0001.

To investigate effective and low-toxicity therapeutic agents for breast cancer metastasis, we devised a systematic repurposing screening of 140 FDA-approved drugs (Figure 1A). Initially, highly metastatic murine TNBC 4T1 cells were individually screened with 140 drugs (Supplementary Table S1) using the cell viability assay (Supplementary Figure S1A–C). The 140 compounds encompass diverse molecular targets and signaling pathways, and the majority hold U.S. FDA approval. Based on their IC_50_ value, the top 35 candidate drugs were selected, and their low-dose IC_20_ concentrations were subsequently validated (Supplementary Figure S1D).

To evaluate the anti-migratory potential of these candidates under low-dose conditions, a high-throughput scratch wound-healing assay was performed using the Incucyte live-cell imaging system (Figure 1B–F). Cisplatin at its IC_20_ concentration served as a positive control, and drugs that more effectively inhibited cell migration compared to cisplatin were considered promising candidates. Among the top 35 candidates, low-dose microtubule inhibitors (paclitaxel and docetaxel) and SRC/Abelson (ABL) dual inhibitors (dasatinib and bosutinib) exhibited strong anti-migratory effects (Figure 1C–F). These effects were also observed in the human TNBC cell line MDA-MB-231. Notably, paclitaxel and docetaxel are already approved for the treatment of metastatic breast cancer [30]. Therefore, dasatinib was selected for further investigation.

In the clonogenic formation assay, dasatinib inhibited 4T1 cell proliferation in a dose-dependent manner (Supplementary Figure S1E). In addition to its anti-migratory effects, dasatinib significantly reduced the invasive capacity of both 4T1 and MDA-MB-231 cells (Figure 1G,H).

Collectively, low-dose dasatinib effectively suppressed cell proliferation, migration, and invasion in vitro, indicating that low-dose dasatinib may enhance therapeutic efficacy against tumor metastasis.

### 3.2. Dasatinib Hinders Breast Cancer Cell Survival and Metastasis

**Figure 2:**
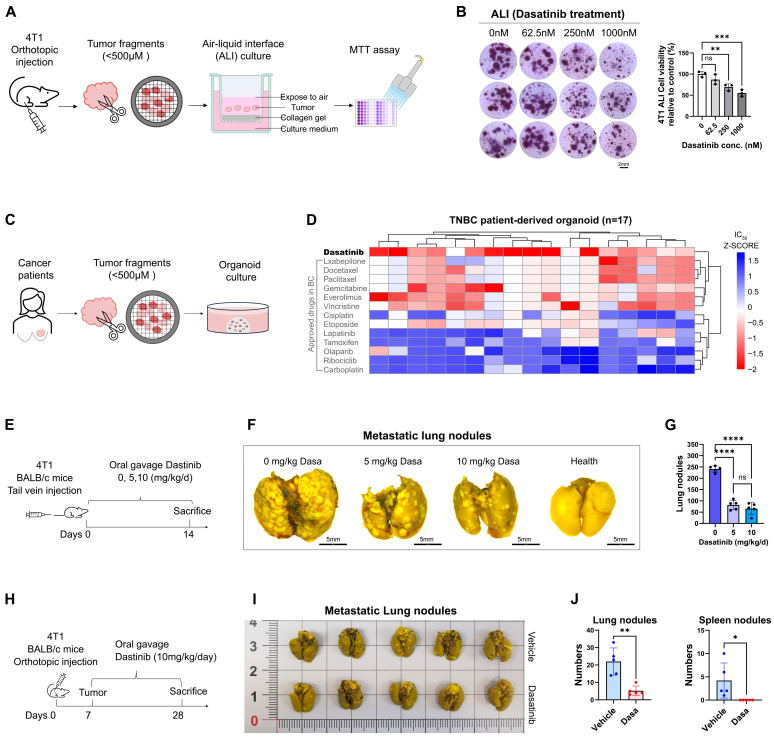
Dasatinib inhibits breast cancer metastasis. (**A**) Schematic illustration of the air–liquid interface (ALI) culture system. Orthotopic 4T1 tumors from BALB/c mice were dissected and cultured under ALI conditions with drug treatment for 7 days. (**B**) Representative images of BALB/c-derived 4T1 tumors treated with varying doses of dasatinib in ALI culture. Cell viability of ALI-cultured organoids assessed by MTT assay following dasatinib treatment. (**C**) Schematic illustration of the patient-derived organoid (PDO) culture process. Human TNBC tumor tissues were dissected and used to generate PDOs for subsequent drug screening. (**D**) Heatmap showing the response of TNBC PDOs to dasatinib and other clinically used breast cancer drugs. (**E**) Schematic of the tail vein injection model. 4T1 cells (2 × 10^5^ cells) were injected into BALB/c mice (n = 5 mice/group) on day 0, followed by daily oral gavage of vehicle or dasatinib for 14 days. (**F**) Representative lung images fixed by Bouin’s solution (scale bar = 5 mm), with metastatic nodules indicated by white foci. (**G**) Quantification of metastatic nodules in the lungs. (**H**) Schematic of the orthotopic implantation model. 4T1 cells (5 × 10^5^) were implanted into the fourth mammary fat pad of BALB/c mice (n = 5 mice/group) on day 0. Dasatinib (10 mg/kg/day) or vehicle was administered via oral gavage from day 7 to 28. (**I**) Representative images of primary tumors and lungs fixed by Bouin’s solution, with metastatic nodules indicated by white foci. (**J**) Quantification of the number of metastatic nodules in the lungs and spleens. Data were represented as mean ± SD. Statistical analysis was determined using a two-tailed *t*-test (two groups). ns, not significant, * *p* < 0.05, ** *p* < 0.01, *** *p* < 0.001, **** *p* < 0.0001.

To explore the therapeutic effect of dasatinib in suppressing breast cancer progression, we employed ex vivo and in vivo approaches, including three-dimensional (3D) air–liquid interface (ALI) culture, patient-derived organoid (PDO) culture, and allograft mouse models. In the ALI model, minced 4T1 tumor tissue from mice was embedded in collagen gel within the inner Transwell chamber, allowing air exposure, integrated tumor growth, and preservation of the natural ECM and immune components to closely mimic the tumor microenvironment (TME) ex vivo [22] (Figure 2A). Dasatinib treatment significantly inhibited 4T1 tumor tissue growth in a dose-dependent manner in samples derived from immunocompetent BALB/c mice (Figure 2B).

Furthermore, in our previous study [31], drug screening on human PDO samples revealed that dasatinib exerted potent cytotoxic effects in 17 TNBC PDOs and demonstrated superior efficacy compared with other clinically approved agents for breast cancer. These findings underscore its potential as a promising therapeutic option for patients with TNBC (Figure 2C,D).

Cancer metastasis is a multistep process in which tumor cells acquire invasive properties, degrade the ECM, intravasate into the circulation, survive transit, disseminate to distant organs, and ultimately colonize secondary sites [10,11,12]. To determine whether dasatinib inhibits lung colonization by breast cancer cells, we employed two in vivo models. In the tail vein model (Figure 2E), which bypasses the primary tumor and focuses on metastatic colonization [32], mice treated with dasatinib led to a dose-dependent reduction in lung metastases (Figure 2F,G). In the orthotopic models (Figure 2H), 4T1 cells were injected into the mammary fat pad of BALB/c mice to recapitulate the full metastasis cascade [33]. Dasatinib treatment significantly reduced the number of metastatic nodules in the lungs and spleens (Figure 2I,J), indicating that dasatinib can inhibit tumor cell metastasis to distant organs.

Taken together, these findings suggest that dasatinib is a promising therapeutic candidate for inhibiting the progression and metastatic spread of breast cancer.

### 3.3. Dasatinib Prevents Metastasis by Disrupting the Actin Cytoskeleton

**Figure 3:**
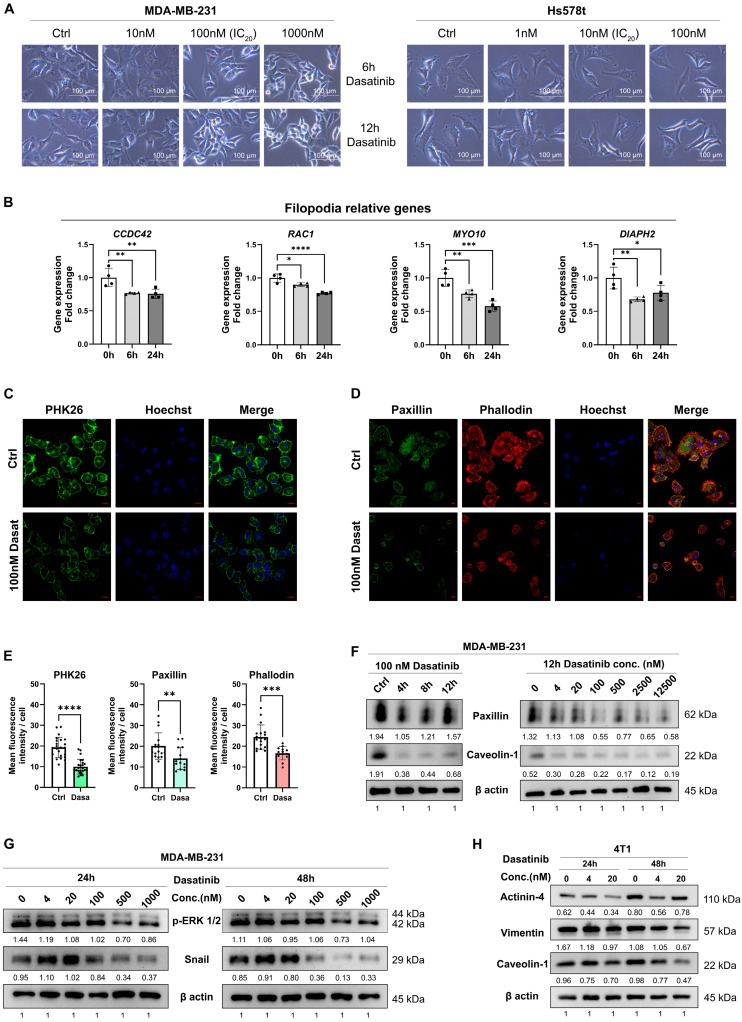
Dasatinib disrupts the cytoskeleton in TNBC cells. (**A**) Representative bright-field images of MDA-MB-231 and Hs578T cells treated with dasatinib (scale bar = 100 μm). (**B**) RNA level of genes associated with filopodia formation following 100 nM dasatinib treatment. Data normalized to *β-actin* and presented as mean ± SD of quadruplicate experiments. Significant differences were determined using one-way ANOVA. * *p* < 0.05, ** *p* < 0.01, *** *p* < 0.001, **** *p* < 0.0001. (**C**) Representative confocal images of PHK26 (green) in MDA-MB-231 cells treated with 100 nM dasatinib for 12 h. Images were captured using a 63× objective confocal microscope (scale bar = 20 μm). (**D**) Representative confocal images of paxillin (green) and phalloidin (F-actin staining, red) in MDA-MB-231 cells treated with 100 nM dasatinib for 12 h. Images were captured using a 63× objective confocal microscope (scale bar = 10 μm). (**E**) Qualification of mean fluorescence intensity per cell of PHK26, paxillin, and phalloidin staining using ImageJ software. ** *p* < 0.01, *** *p* < 0.001, **** *p* < 0.0001. (**F**–**H**) Western blot analysis of protein level following dasatinib treatment. Qualified data were normalized to the reference control of β-actin using ImageJ software.

To examine dasatinib’s effect on cell morphology, we observed structural changes in TNBC cell lines. Dasatinib significantly inhibited lamellipodia and filopodia formation in MDA-MB-231 and Hs578T cells (Figure 3A), which are critical structures for cell migration and invasion. Consistently, dasatinib downregulated key cytoskeletal regulators (*CDC42*, *RAC1*, *MYO10*, *DIAPH2*) involved in filopodia formation (Figure 3B), indicating compromised cytoskeletal integrity and diminished motility capacity.

To further validate these observations, we employed immunofluorescence and Western blot targeting specific structural markers. Membrane integrity was assessed using PKH26 (fluorescent membrane lipid dye) and anti-caveolin-1 (*CAV1*; lipid raft scaffold protein antibody), while cytoskeletal architecture was analyzed via phalloidin (F-actin probe) and anti-paxillin (focal adhesion protein antibody). Dasatinib treatment reduced membrane lipid rafts and cell spreading area and diminished cytoskeletal protrusions (Figure 3C–E). Phalloidin staining further demonstrated that dasatinib induced cytoskeletal disorganization, with cells transitioning from spindle-shaped morphology to rounded or punctate configurations. Concomitant reduction in focal adhesion protein paxillin was also observed. In addition, Western blot analysis confirmed dose- and time-dependent decreases in paxillin and caveolin-1 protein levels (Figure 3F). Moreover, dasatinib suppressed epithelial–mesenchymal transition (EMT) markers (Snail, Vimentin) and phosphorylated extracellular signal-regulated kinases (p-ERK1/2), indicating inhibition of EMT and MAPK signaling pathways (Figure 3G,H).

Overall, these findings demonstrate that dasatinib disrupts actin cytoskeletal dynamics, impairs cell motility, and attenuates downstream oncogenic pathways (EMT/MAPK), ultimately reducing metastatic potential in aggressive breast cancers.

### 3.4. Dasatinib Exhibits High Sensitivity in the Basal B Breast Cancer with High ETS1 Expression

Breast cancer cell lines were classified into luminal A (LA), luminal B (LB), HER2-positive (H), and TNBC of basal A (BaA) and basal B (BaB) subtypes [5,6]. While our initial experiments focused on TNBC cell lines of murine 4T1 and human MDA-MB-231, the broader applicability of dasatinib across breast cancer subtypes remained unclear.

**Figure 4:**
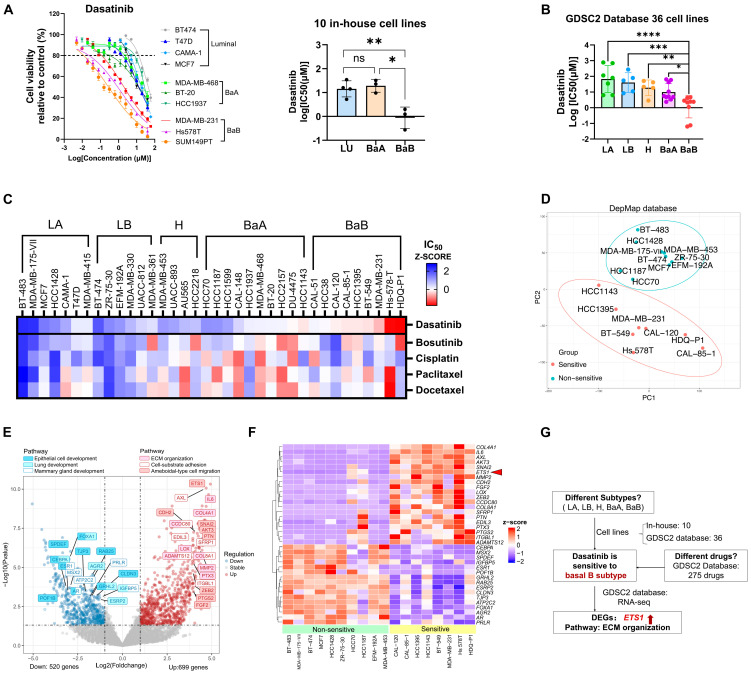
Dasatinib exhibits high sensitivity in basal B breast cancer with high ETS1 activity. (**A**) Cell viability of 10 in-house human breast cancer cell lines treated with dasatinib. Breast cancer cell lines include luminal (BT474, T47D, MCF7), basal A (MDA-MB-468, BT-20, HCC1937) and basal B (MDA-MB-231, Hs578T, SUM149PT). Data: mean ± SD. Significance: one-way ANOVA. ns, not significant, * *p* < 0.05, ** *p* < 0.01. (**B**) IC_50_ value across 36 human breast cancer cell lines from the GDSC2 database treated with dasatinib. Each dot represents the IC_50_ value for one cell line. Abbreviation: LA, luminal A; LB, luminal B; H, HER2-enriched; BaA, basal A; BaB, basal B. Data: mean ± SD. Significance: one-way ANOVA. * *p* < 0.05, ** *p* < 0.01, *** *p* < 0.001, **** *p* < 0.0001. (**C**) IC_50_ value across 36 cell lines from the GDSC2 database treated with bosutinib, cisplatin, paclitaxel, and docetaxel. A heatmap represents the cell viability of the Z-score of IC_50_ in different cell lines. (**D**) Principal Component Analysis (PCA) of breast cancer cell lines based on RNA-seq data from the DepMap database, distinguishing two groups of dasatinib-sensitive (IC_50_ < 2.85 µM) and non-sensitive (IC_50_ > 50 µM) cell lines. (**E**) Volcano plot showing differentially expressed genes (DEGs) involved in enriched pathways between dasatinib-sensitive versus non-sensitive cell lines. Downregulated genes (blue; n = 520) and upregulated genes (red; n = 699) were identified using a fold change > 1.5, *p*-value < 0.05. (**F**) Heatmap of DEGs associated with enriched pathways in different cell lines. (**G**) Schematic summary of dasatinib sensitivity. Red arrow indicates the higher expression of *ETS1* in dasatinib sensitive cells when comared to the non-sensitive ones.

To address this, we assessed dasatinib sensitivity across 10 in-house human breast cancer cell lines, including luminal (BT474, T47D, MCF7), basal A (MDA-MB-468, BT-20, HCC1937), and basal B (MDA-MB-231, Hs578T, SUM149PT). Notably, dasatinib exhibited increased sensitivity in basal B subtype cell lines (Figure 4A). Next, we extended our analysis to 36 cell lines from the Genomics of Drug Sensitivity in Cancer 2 (GDSC2) database [23], which confirmed that dasatinib displayed selective potency in basal B cells, with significantly lower IC_50_ values compared to other subtypes (Figure 4B). To determine whether this subtype-specific sensitivity was unique to dasatinib, we analyzed IC_50_ values for 275 drugs across the same 36 cell lines using the GDSC2 database. Our integrated analysis revealed that dasatinib’s selective potency in basal B cells was unique, as other compounds exhibited more variable activity profiles across subtypes (Figure 4C and Figure S2A). Notably, both dasatinib and bosutinib are dual SFK/ABL inhibitors that suppress cell migration, but they differ in subtype preferences. After analyzing published kinome profiling datasets to compare their kinase binding affinities [34], we found that dasatinib has a significantly higher affinity for SFKs, while bosutinib showed slightly higher affinity for ABL kinases (Supplementary Figure S2B).

To further elucidate the molecular characteristics underlying dasatinib sensitivity, we compared gene expression profiles between dasatinib-sensitive (IC_50_ < 2.85 µM) and non-sensitive (IC_50_ > 50 µM) cell lines. Principal component analysis (PCA) separated the two groups within a 95% confidence interval (Figure 4D), indicating distinct transcriptomic profiles. Differentially expressed genes (DEGs) were visualized in a volcano plot comparing dasatinib-sensitive versus non-sensitive groups (Figure 4E) and in a heatmap displaying expression patterns across individual cell lines (Figure 4F). Analysis revealed significant downregulation of luminal markers (*ESR1*, *FOXA1*, *SPDEF*), whereas ETS proto-oncogene 1 (*ETS1*), a transcription factor associated with TNBC, was significantly upregulated [5,35]. The downregulated genes (*ESR1*, *AR*, *SPDEF*, and *FOXA1*) participate in hormone receptor signaling and epithelial integrity in breast cancer [36,37,38]. In contrast, upregulated genes in sensitive cell lines were enriched in pathways related to EMT process (*CDH2*, *SNAI2*, *ZEB2*, *IL6*) [39,40], ECM remodeling (*MMP2*, *LOX*, *COL4A1*, *COL8A1*) [10], cancer stemness (*PTGS2*/*COX-2*) [41,42], angiogenesis and cell migration (*FGF2*, *EDIL3*, *PTN*) [43,44,45], and drug resistance and metastasis (*ETS1*, *AXL*, *ADAMTS12*) [46,47,48]. Gene Ontology (GO) enrichment analysis corroborated these findings. Pathways enriched among upregulated genes in dasatinib-sensitive cell lines included ECM organization, cell–substrate adhesion, and cell migration (Figure 4E and Figure S2C). In contrast, downregulated pathways were associated with epithelial cell functions, mammary gland differentiation, and lung development (Figure 4E and Figure S2C). These pathways are critically involved in tumor progression and underpin the aggressive phenotype of dasatinib-sensitive cell lines.

Collectively, the integrated analysis of 10 in-house and 36 publicly available breast cancer cell lines revealed dasatinib sensitivity as specific to the basal B subtype, which is unique among 275 compounds screened (Figure 4G and Figure S2F,G, Table S2). Our findings indicate that dasatinib exhibits preferential activity against aggressive TNBC cell lines characterized by high ETS1 expression, with sensitivity driven by ECM pathway activation.

### 3.5. Dasatinib Modulates ECM Organization and Suppresses ETS1 and MMP3 Expression

**Figure 5:**
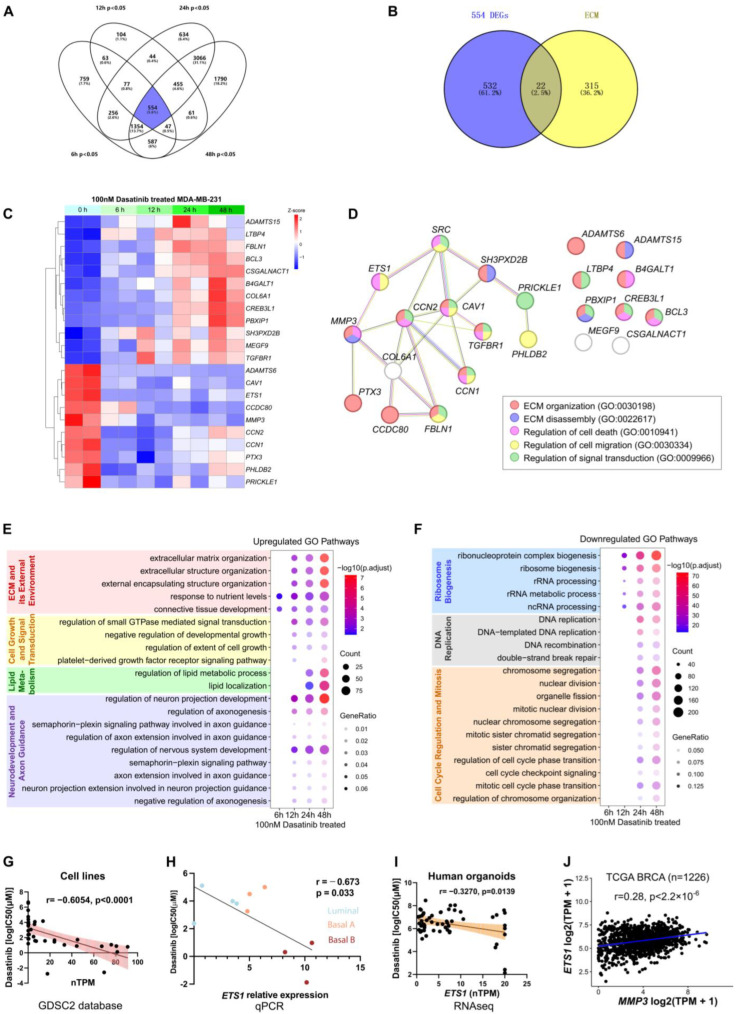
Transcriptomic analysis of MDA-MB-231 cells following dasatinib treatment. (**A**) Venn diagram of differentially expressed genes (DEGs; *p* < 0.05) in MDA-MB-231 cells treated with 100 nM dasatinib for 6, 12, 24, and 48 h, compared to untreated controls (0 h). (**B**) Overlap of DEGs (n = 554) at four time points and genes associated with the extracellular matrix (ECM) pathway (GO:0030198). (**C**) Heatmap showing expression profiles of 22 ECM-related genes in MDA-MB-231 cells treated with 100 nM dasatinib across four time points. (**D**) Protein–protein interaction network illustrating the association between SRC and the 22 ECM-related genes. (**E**) Top 20 commonly upregulated Gene Ontology (GO) pathways across four time points under dasatinib treatment. (**F**) Top 20 commonly downregulated GO pathways across four time points under dasatinib treatment. (**G**) Correlation between dasatinib sensitivity (log IC_50_ [µM]) and *ETS1* expression (nTPM) across 36 human breast cancer cell lines from the GDSC2 database. Each dot represents one cell line. Correlation was assessed using the Pearson correlation in GraphPad Prism. (**H**) Correlation between dasatinib sensitivity (log IC_50_ [µM]) and *ETS1* mRNA expression measured by qPCR across 10 in-house breast cancer cell lines. (**I**) Correlation between dasatinib sensitivity (log IC_50_ [µM]) and *ETS1* expression (nTPM) across 76 human organoids. Each dot represents one organoid. Correlation was assessed using the Pearson correlation coefficient. (**J**) Correlation between MMP3 and ETS1 mRNA expression in breast cancer (BRCA) samples (n = 1226) from The Cancer Genome Atlas (TCGA) database. Correlation was assessed using the Pearson correlation coefficient.

To elucidate the molecular mechanisms by which dasatinib inhibits breast cancer progression, we conducted bulk RNA sequencing (RNA-seq) on MDA-MB-231 cells treated with dasatinib for 6, 12, 24, and 48 h, respectively. Differential expression analysis identified 554 genes consistently altered (*p* < 0.05) across all time points (Figure 5A). Given the enrichment of ECM organization pathways in both dasatinib-sensitive cell lines and treated samples, we focused on ECM-related genes (GO:0030198) and identified 22 overlapping genes (Figure 5B), whose expression profiles are visualized via a heatmap (Figure 5C).

As dasatinib is a known SRC kinase inhibitor, we utilized the Search Tool for the Retrieval of Interacting Genes/Proteins (STRING) database to construct a protein–protein interaction (PPI) network linking SRC to the 22 ECM-related genes (Figure 5D). *ETS1* emerged as a central node associated with dasatinib sensitivity (Figure 4E), prompting further investigation into the SRC-ETS1-MMP3 regulatory axis.

GO analysis revealed dynamic biological processes over time. Dasatinib treatment upregulated pathways associated with ECM organization and small GTPase-mediated signaling, suggesting drug-induced cellular stress through structural remodeling and enhanced motility. Concurrent enrichment of lipid metabolic reprogramming indicated increased energy demands and membrane alterations. Furthermore, enrichment of genes involved in cell motility overlapped with axon guidance pathways, reflecting shared activation of neurodevelopmental pathways (Figure 5E). Conversely, downregulated genes were enriched in ribosome biogenesis and DNA replication, indicative of suppressed proliferation and a potential shift toward cellular quiescence or a stressed response state (Figure 5F).

Among 36 breast cancer cell lines, *ETS1* expression negatively correlated with dasatinib IC_50_ values (r = −0.6054, *p* < 0.0001) (Figure 5G). Similarly, this relationship was recapitulated in our qPCR data, where *ETS1* expression also inversely correlated with dasatinib IC_50_ values (r = −0.673, *p* = 0.033) (Figure 5H). Moreover, in our breast cancer PDO samples [31], *ETS1* expression was negatively associated with dasatinib (r = −0.3270, *p* = 0.0139) (Figure 5I). These findings suggest that dasatinib exhibits pronounced efficacy in *ETS1*-high samples. Notably, previous studies have reported increased *ETS1* mRNA levels in invasive breast cancer [49]. As a transcription factor, ETS1 regulated the expression of *MMP3* [50,51,52]. Consistently, a strong positive correlation between *ETS1* and *MMP3* mRNA expression was found in The Cancer Genome Atlas (TCGA) dataset (n = 1226; *p* < 2.2 × 10^−16^) (Figure 5J). These findings suggest that elevated expression of *ETS1* and *MMP3* may be associated with increased sensitivity to dasatinib treatment.

**Figure 6:**
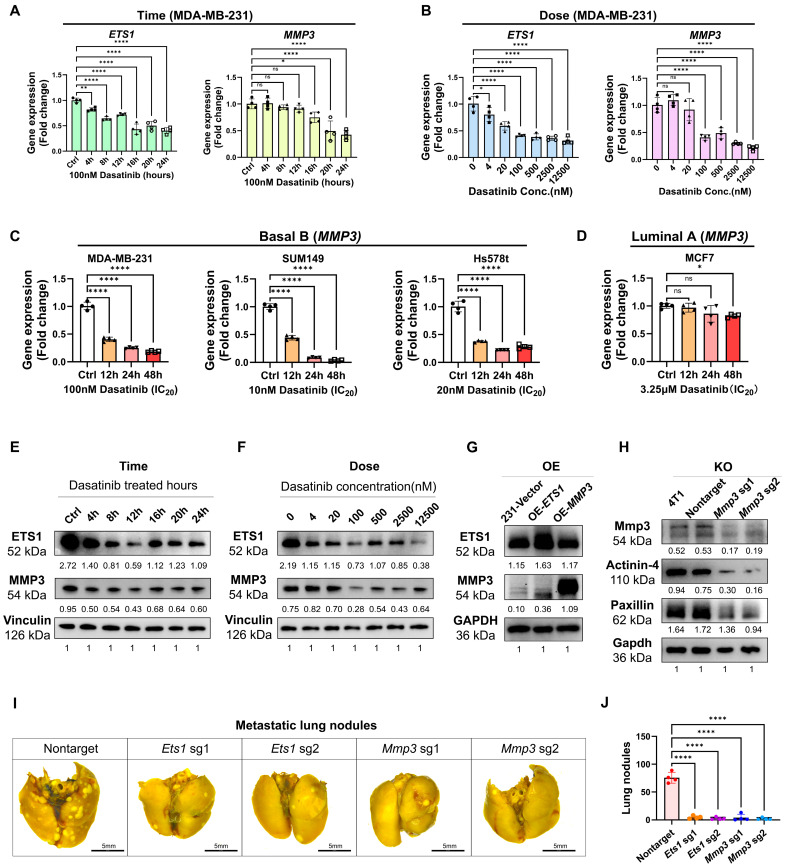
Dasatinib suppressed ETS1 and MMP3 expressions. (**A**–**D**) *ETS1* and *MMP3* mRNA expression levels following dasatinib treatment, measured by RT-qPCR. Data normalized to *β-actin* and presented as mean ± SD of quadruplicate experiments. (**E**,**F**) Western blot analysis of ETS1 and MMP3 protein levels in MDA-MB-231 cells. Cells were treated with dasatinib at indicated durations with 100 nM dasatinib or graded concentrations for 12 h. Protein levels were quantified using ImageJ and normalized to vinculin. (**G**) Western blot analysis of *ETS1* and *MMP3*-overexpression (OE) in MDA-MB-231 cells. Protein levels were quantified using ImageJ and normalized to GAPDH. (**H**) Western blot analysis of *Mmp3*-knockout (KO) in 4T1 cells. Protein levels were quantified using ImageJ and normalized to GAPDH. (**I**) Represented lung images fixed by Bouin’s solution (scale bar = 5 mm) from BALB/c mice following tail vein injection of 4T1-KO cells at the endpoint of 14 days. (**J**) Quantification of metastatic lung nodules. Data were represented as mean ± SD. Statistical analysis was determined using one-way ANOVA. ns, not significant, * *p* < 0.05, ** *p* < 0.01, **** *p* < 0.0001.

Subsequently, we sought to verify the roles of ETS1 and MMP3 in dasatinib-mediated inhibition of breast cancer progression. In MDA-MB-231 cells, dasatinib downregulated both ETS1 and MMP3 expression in dose- and time-dependent manners (Figure 6A,B). Similar reductions in MMP3 expression were observed in other basal B subtype cell lines of SUM149 and Hs578T, but not in the luminal A subtype MCF7 (Figure 6C,D), confirming subtype-specific regulation. Concordantly, dasatinib treatment resulted in a dose- and time-dependent reduction in ETS1 and MMP3 protein levels (Figure 6E,F).

To investigate the functional role of ETS1 and MMP3 in the context of dasatinib treatment in aggressive TNBC, we generated stable cell lines overexpressing (OE) ETS1 or MMP3 using the PLV3 plasmid and knockout (KO) cell lines using the CRISPR-Cas9 V2 system. ETS1-OE increased MMP3 protein levels, whereas MMP3-OE did not alter ETS1 levels (Figure 6G), suggesting the notion that ETS1 acts upstream of MMP3. Furthermore, MMP3-KO reduced the expression of ECM components such as Actinin-4 and Paxillin (Figure 6H).

To further assess the roles of ETS1 and MMP3 in metastasis, Ets1-KO and Mmp33-KO 4T1 cells (Figure 6H and Figure S2E) were intravenously injected into BALB/c mice via the tail vein. Depletion of either MMP3 or ETS1 significantly reduced the lung metastatic burden compared to controls, with no significant body weight loss observed (Figure 6I,J).

In summary, these findings underscore the pivotal roles of ETS1 and MMP3 in dasatinib-induced ECM remodeling and suppression of TNBC metastasis, positioning the SRC/ETS1/MMP3 signaling axis as a promising therapeutic target.

### 3.6. Dasatinib Potentiates Anti-Tumor Effects via the Immune Microenvironment

**Figure 7:**
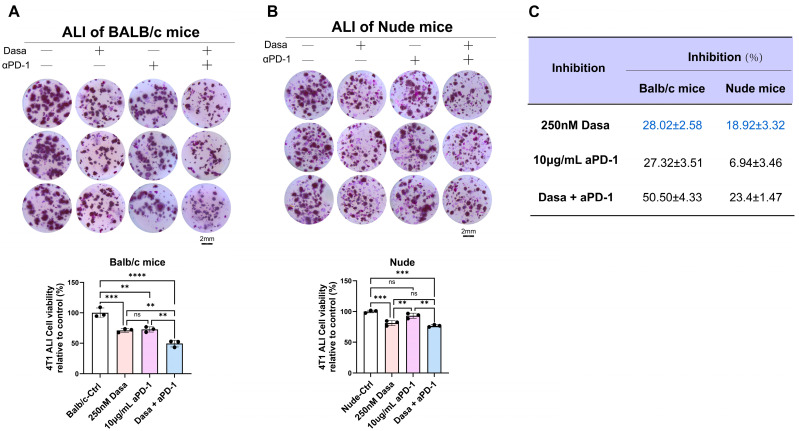
Dasatinib enhances anti-tumor efficacy by modulating the immune microenvironment. (**A**) Air–liquid interface (ALI) culture of 4T1 tumors derived from BALB/c mice treated with dasatinib, anti-PD-1 antibody, or the combination (scale bar = 2 mm). Representative images and quantification of organoid viability via MTT assay were shown. (**B**) ALI culture of 4T1 tumors derived from nude mice treated with dasatinib, anti-PD-1 antibody, or the combination (scale bar = 2 mm). Representative images and corresponding quantification of organoid viability measured by MTT assay were presented. (**C**) Comparative inhibition of tumor viability by single or combination treatment with 250 nM dasatinib and 10 μg/mL anti-PD-1 antibody in ALI cultures derived from BALB/c or nude mice. Data are presented as mean ± SD. Statistical analysis: One-way ANOVA. ns, not significant, ** *p* < 0.01, *** *p* < 0.001, **** *p* < 0.0001.

ECM remodeling is generally associated with immune system involvement [53]. To investigate whether the anti-tumor effect of dasatinib in breast cancer requires immune participation, we utilized ALI culture from both immunodeficient BALB/c mice and immunocompetent nude mice. The results showed that dasatinib exerted stronger cytotoxic effects in 4T1 tumors derived from immunocompetent mice than from immunodeficient mice (Figure 7A–C). Given that anti-PD-1 antibodies represent a current focus of immunotherapy, we further evaluated their combined efficacy. Both dasatinib and anti-PD-1 monotherapies suppressed tumor growth, while their combination produced greater inhibitory effects than either agent alone. In contrast, in tumors from immunodeficient mice, no significant difference was observed between dasatinib monotherapy and the combination with anti-PD-1 antibodies.

Collectively, these findings support the combination of dasatinib and anti-PD-1 immunotherapy as a promising therapeutic strategy for enhancing anti-tumor responses. Moreover, they suggest that dasatinib may potentiate the therapeutic outcomes by modulating the immune microenvironment.

## 4. Discussion

Owing to the absence of well-defined molecular targets, the therapeutic landscape for TNBC/basal-like breast cancer remains especially challenging, necessitating the development of alternative therapeutic strategies [54]. Among 140 compounds screened, our study identified dasatinib as a promising therapeutic agent for TNBC/basal-like breast cancer. Although dasatinib has demonstrated substantial efficacy in multiple preclinical models [16,17,55], its clinical performance in unselected patients with metastatic breast cancer has been largely disappointing [56,57]. This discrepancy raises the question of whether dasatinib exhibits selective efficacy in specific breast cancer subtypes.

Lehmann et al. classified TNBC into six molecular subtypes based on gene expression profiles: basal-like 1 (BL1), basal-like 2 (BL2), mesenchymal (M), mesenchymal stem-like (MSL), immunomodulatory (IM), and luminal androgen receptor (LAR) [58]. The BL1 and BL2 subtypes are characterized by elevated expression of genes involved in the cell cycle and DNA damage response, rendering them responsive to cisplatin treatment [58]. In contrast, the M and MSL subtypes, which are enriched in the EMT and ECM-related pathways, exhibited preferential sensitivity to dasatinib [58]. Based on transcriptomic profiles, the M and MSL subtypes closely resemble the basal B subtype, characterized by a more mesenchymal-like morphology, enhanced stemness, and a highly invasive phenotype [5,6]. These features may underline the selective vulnerability of these subtypes to dasatinib, highlighting the importance of molecular stratification in optimizing the therapeutic outcomes.

Similarly, through integrated analysis of 10 in-house human breast cancer cell lines and 36 external cell lines, our study identified dasatinib sensitivity as uniquely associated with the basal B subtype among 275 compounds screened. Previous studies have shown that basal-type and post-EMT breast cancer cell lines are particularly sensitive to dasatinib, with elevated expression of genes such as moesin (*MSN*), caveolin-1 (*CAV1*), and yes-associated protein-1 (*YAP1*), which are associated with the SRC signaling pathway [59]. In our study, gene expression profiling of dasatinib-sensitive versus non-sensitive cells (in the absence of drug treatment) revealed significantly higher levels of *CAV1* (Log2FC = 2.78, *p*-value = 1.2 × 10^−9^) and *MSN* (Log2FC = 2.71, *p*-value = 6.62 × 10^−8^) in dasatinib-sensitive breast cancer cell lines. Notably, *ETS1* emerged as the most upregulated gene (Log2FC = 4.92, *p*-value = 4.78 × 10^−11^), and its expression was significantly reduced following dasatinib treatment, suggesting that *ETS1* is a key determinant of subtype-specific sensitivity to dasatinib.

*ETS1* is a nuclear transcription factor implicated in cancer progression and metastasis [50,51]. Elevated *ETS1* expression has been observed in TNBC and is positively correlated with SRC family kinase activity. Dasatinib has been shown to dramatically suppress *ETS1* expression [52]. Additionally, guanylate-binding protein 5 (GBP5) promotes glioblastoma malignancy through the SRC/ERK1/2/MMP3 pathway [60], implicating *ETS1* and *MMP3* as downstream effectors of SRC signaling. In addition, phosphorylation of both ERK1 and ERK2 is significantly inhibited by dasatinib, demonstrating that the classic MAPK signaling is involved in the dasatinib-mediated inhibition of breast cancer growth and metastasis. Our findings indicate that dasatinib modulates ECM organization and suppresses both *ETS1* and *MMP3*. As a transcription factor, *ETS1* directly binds the *MMP3* promoter [50,51], and its inhibition leads to reduced *MMP3* expression.

*MMP3* is a zinc-dependent endopeptidase involved in ECM degradation and remodeling [61,62]. Aberrant *MMP3* expression promotes epithelial malignancy [63], is elevated in invasive breast cancer, and is associated with poor prognosis [64]. Overexpression of *MMP3* activates canonical Wnt signaling, drives stem cell expansion, disrupts epithelial homeostasis, and contributes to tumorigenesis [65]. However, clinical trials using MMP inhibitors or antibodies have yielded disappointing results due to toxicity, poor specificity, and metabolic instability [61,66]. These challenges underscore the need for alternative strategies to regulate *MMP3*. A recent study demonstrated that pharmacological inhibition or genetic downregulation of *KDM6A* attenuated *MMP3* expression and suppressed TNBC brain metastasis [67]. Consistent with these findings, our data showed that dasatinib downregulates *MMP3* and that *MMP3* knockout impairs metastatic potential, suggesting dasatinib as a viable therapeutic strategy to target *MMP3* in breast cancer.

EMT plays a crucial role in cancer metastasis [68]. During EMT, epithelial cells lose apical–basal polarity and acquire front–rear polarity, accompanied by cytoskeletal reorganization, cell shape changes, junctional disruption, and detachment from the ECM [8,9]. This process is driven by RhoA GTPase signaling, which promotes actin cytoskeleton remodeling, contractility, and the formation of membrane protrusions such as filopodia and invadopodia [8,9]. Our results demonstrate that dasatinib reduces expression of EMT markers (vimentin, snail), changes the mesenchymal morphology, and disrupts filopodia formation and actin cytoskeletal dynamics.

During migration, actin polymerization drives membrane ruffling, lamellipodia, and filopodia extension at the leading edges [69]. Actin network remodeling through depolymerization and debranching facilitates lamellipodial dynamics and cell propulsion [70,71]. Invadopodia are F-actin–rich structures at the cell and ECM interface that release proteases to degrade ECM components, enabling rapid and irreversible matrix remodeling and promoting invasion [69,72]. Dasatinib’s inhibition of cytoskeletal remodeling likely contributes to its anti-metastatic effect. Notably, dasatinib treatment significantly reduced the expression of two isoforms of Paxillin (Figure 3F). These isoforms of Paxillin may play distinct functions in cell adhesion regulation of cancer cells [73]. And their roles in dasatinib-mediated cell skeleton and ECM remodeling deserve further study in the future.

Although dasatinib monotherapy has shown limited clinical efficacy, combination therapies may enhance therapeutic outcomes [56,57]. Previous studies have reported that dasatinib reduced tumor growth and increased CD8 T cell infiltration in various cancers, including B16.OVA melanoma, 1956 sarcoma, MC38 colon, and 4T1 breast carcinoma [74]. These findings highlight the potential of combining dasatinib with immunotherapies. Using a 3D-ALI culture model that preserves native ECM and immune components ex vivo [22], we observed that dasatinib exerted significantly greater inhibitory effects on tumor tissues derived from immunocompetent BALB/c mice compared to those from immunodeficient nude mice. This suggests that host immune status modulates dasatinib efficacy.

Immune checkpoint blockade targeting PD-1 has demonstrated clinical success across multiple cancer types [75]. In our ALI model, the combination of dasatinib and anti-PD-1 antibody significantly suppressed tumor tissue growth in BALB/c mice, but not in nude mice. Similarly, dasatinib combined with anti-PD-1 therapy has shown enhanced efficacy in acute lymphocytic leukemia (ALL) [76], colon cancer [77,78], and non-small cell lung cancer (NSCLC) [79]. Recent studies have shown that dasatinib can reprogram cancer-associated fibroblasts (CAFs) and remodel the ECM [80,81]. Furthermore, nanomedicine formulations combining dasatinib with anti-PD-1 agents have demonstrated improved tumor penetration and enhanced immune responsiveness [82,83]. These data support further investigation into the mechanisms underlying dasatinib and anti-PD-1 combination treatment in breast cancer and its translation into clinical strategies.

In summary, our study identified dasatinib as a promising anti-metastatic agent among 140 screened compounds. Mechanistically, dasatinib inhibits actin cytoskeletal reorganization and filopodia formation—key drivers of cell migration. It also suppresses *ETS1* and *MMP3* expression, leading to ECM remodeling and reduced metastatic potential. These findings help explain the disconnect between dasatinib’s preclinical success and its limited efficacy in unselected clinical populations. Furthermore, we identify ETS1-high basal B TNBC subtypes as particularly responsive to dasatinib, providing a precision oncology framework for patient stratification. Moreover, combination therapy with anti-PD-1 antibodies may further enhance dasatinib’s efficacy by modulating the immune microenvironment.

While our findings offer novel insights into dasatinib-mediated ECM remodeling and metastasis suppression in breast cancer, several limitations remain. Our current experimental models (2D/3D cultures, xenografts) do not fully recapitulate the systemic complexity of human physiology, particularly the immunosuppressive interplay between tumors and the host immune system. Additionally, the signaling pathways linking ECM modeling (via *ETS1*/*MMP3* suppression) to therapeutic outcomes remain incompletely characterized. These limitations underscore the need for future studies to better define ECM–TME interactions and their implications for targeted therapy.

## 5. Conclusions

In conclusion, our study presents several key findings: (1) Dasatinib emerged as a promising anti-metastatic agent from a screen of 140 compounds. (2) Basal B subtype breast cancer cells were particularly sensitive to dasatinib, with *ETS1* emerging as a key modular determinant of this subtype-specific response. (3) Dasatinib disrupted actin cytoskeletal organization and impaired filopodia formation, thereby reducing cellular motility and migratory capacity. (4) Dasatinib suppressed *ETS1* and *MMP3* expression, leading to ECM remodeling and inhibition of cancer cell invasion and metastasis. (5) Combination therapy of dasatinib and anti-PD1 antibody may enhance therapeutic efficacy by modulating the tumor microenvironment and immune response.

## Figures and Tables

**Table 1:** The sequence of sgRNA.

Gene	Name	Direction	Sequence
Human *ETS1*	HGLibA_15640	sg1	AGAGTCGGCTTGAGATCGA
	HGLibA_15641	sg2	TGGAAACCACAGTTCATTCG
Human *MMP3*	HGLibA_29533	sg1	GCATGGGCCAAAACATTTCC
	HGLibA_29534	sg2	GTTCTGAAGTGACCAACATC
Mouse *Ets1*	MGLibA_16711	sg1	CAGAAACCCACGTCCGGGAC
	MGLibA_16712	sg2	CTTACTGATGAAGTAATCCG
Mouse *Mmp3*	MGLibA_31582	sg1	ACTTTGACGATGATGAACGA
	MGLibA_31583	sg2	AATAGGTACCAACCTATTCC

**Table 2:** The sequence of qRT-PCR primers.

Primers	Direction	Sequence
Human *18S*	Forward	AGTCCCTGCCCTTTGTACACA
	Reverse	CGATCCGAGGGCCTCACTA
Human *MMP3*	Forward	CAGGCTTTCCCAAGCAAATAG
	Reverse	CTCCAACTGTGAAGATCCAGTAA
Human *ETS1*	Forward	GATAGTTGTGATCGCCTCACC
	Reverse	GTCCTCTGAGTCGAAGCTGTC
Mouse *Actb*	Forward	GGCTGTATTCCCCTCCATCG
	Reverse	CCAGTTGGTAACAATGCCATGT
Mouse *Mmp3*	Forward	ACATGGAGACTTTGTCCCTTTTG
	Reverse	TTGGCTGAGTGGTAGAGTCCC
Mouse *Ets1*	Forward	GACCTCTTTATCCACTCTGGGA
	Reverse	GTGGTCCTTTCTCCTACGATCA

## Data Availability

The RNA-seq data generated in this study are deposited in the NCBI GEO repository: GSE308797.

## Supplementary Materials

The following supporting information can be downloaded at https://www.mdpi.com/article/10.3390/biomedicines13122888/s1, Supplementary Table S1: The information on 140 drugs. Supplementary Table S2: GDSC2 database information. Supplementary Figure S1: Cell viability test of 140 FDA-approved drugs. Supplementary Figure S2: Dasatinib exhibits high sensitivity to basal B cells.

## Abbreviations

The following abbreviations are used in this manuscript:

ALI	air–liquid interface
ECM	extracellular matrix
EMT	epithelial–mesenchymal transition
ETS1	ETS proto-oncogene 1
TME	Tumor microenvironment
TNBC	triple-negative breast cancer
PD-1	anti-programmed cell death protein-1
PDO	patient-derived organoid
MMP3	matrix metalloproteinase-3
