# Supplementary material for: Dasatinib Inhibits Basal B Breast Cancer Through ETS1-Mediated Extracellular Matrix Remodeling
Source: Biomedicines. 2025 Nov 26;13(12):2888. doi: 10.3390/biomedicines13122888 (PMC12730708; doi:10.3390/biomedicines13122888)
Supplement: Supplementary file 1 [file biomedicines-13-02888-s001.zip › Supplementary Figure S1.pdf]

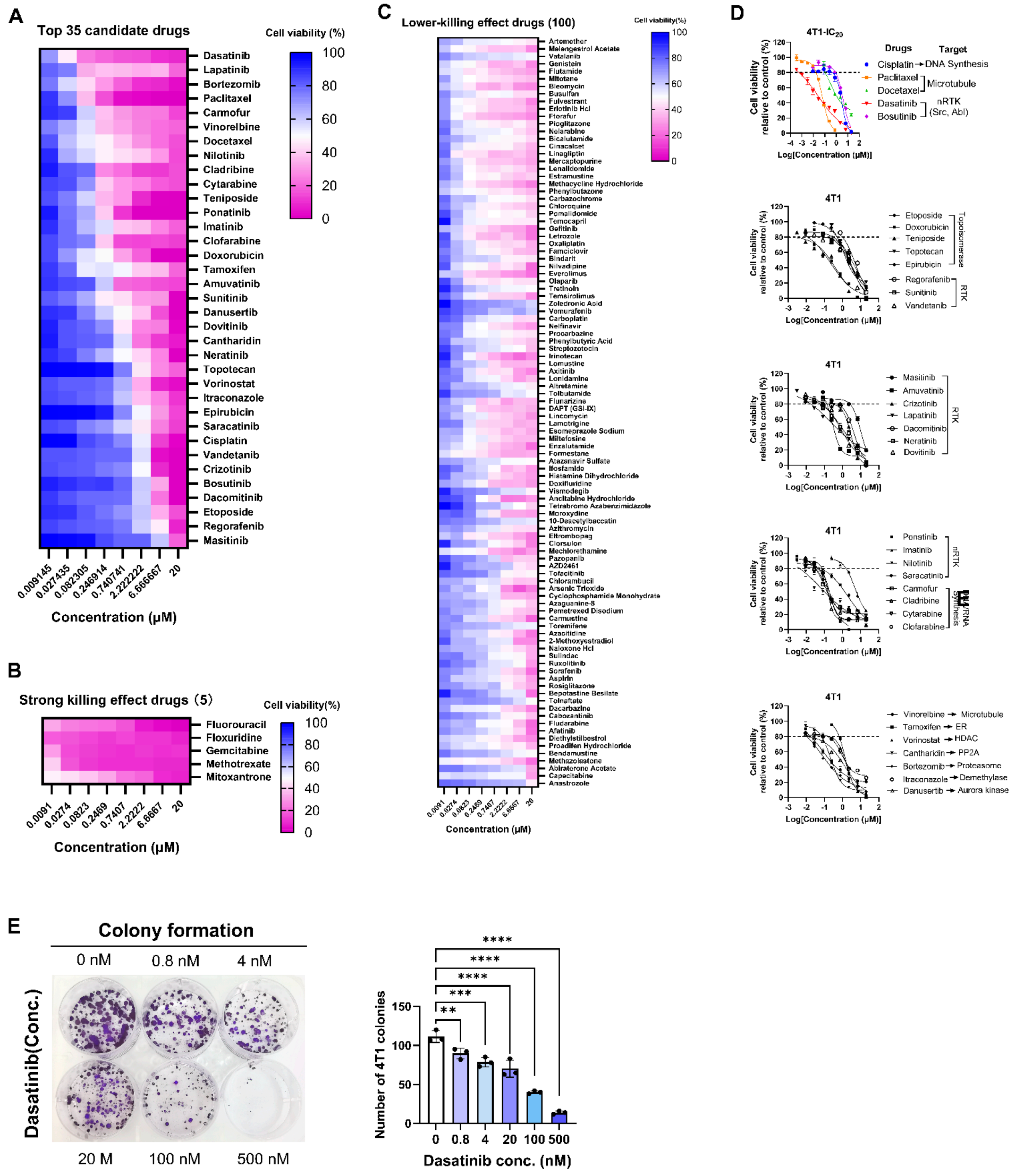

Supplementary Figure S1. Cell viability test of 140 FDA-approved drugs

(**A-C**) Drug screening results. 4T1 cells were exposed to 140 drugs in a threefold serial dilution (20μM-9nM) in 384-well plates. Cell viability was assessed using the AlamarBlue assay from triplicate experiments and represented as a heatmap: (A) top 35 candidate drugs; (B) 5 potent cytotoxic agents demonstrating <50% viability at 9 nM; (C) 100 drugs with reduced cytotoxic activity maintaining >20% viability at 20μM.

(**D**) Low concentration (IC_{20}) efficacy of the top 35 candidate drugs. 4T1 cells were incubated with 35 candidate drugs at indicated concentrations in 96-well plates for 48h, and cell viability was assessed using the AlamarBlue assay. The dotted line indicated 80% cell viability (IC_{20}) relative to the control. Abbreviations: RTK, receptor tyrosine kinase; nRTK, non-receptor tyrosine kinase; ER, estrogen receptor; HDAC, histone deacetylase; PP2A, Protein phosphatase 2.

(**E**) Colony formation assay of 4T1 cells treated with dasatinib. Representative images and quantification from triplicate experiments were shown.
