# Supplementary material for: Dasatinib Inhibits Basal B Breast Cancer Through ETS1-Mediated Extracellular Matrix Remodeling
Source: Biomedicines. 2025 Nov 26;13(12):2888. doi: 10.3390/biomedicines13122888 (PMC12730708; doi:10.3390/biomedicines13122888)
Supplement: Supplementary file 1 [file biomedicines-13-02888-s001.zip › Supplementary Figure S2.pdf]

A

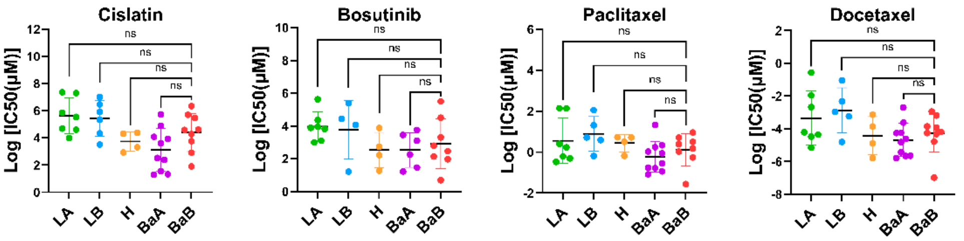

B

Kinases activity

Types	Targets	Dasatinib	Bosutinib
SFK	SRC	3.52	6.05
	LYN	0.43	2.13
	FRK	-0.88	29.2
	LCK	0.31	1.88
	HCK	0.79	11.88
	FGR	1.7	11.49
	FYN	1.96	29.42
	BLK	3.04	20.73
	BRK	4.59	93.08
	YES1	1.94	7.29
	SRMS	16.98	90.65
ABL	ABL1	2.77	1.8
	ABL2	2.74	2.19

C

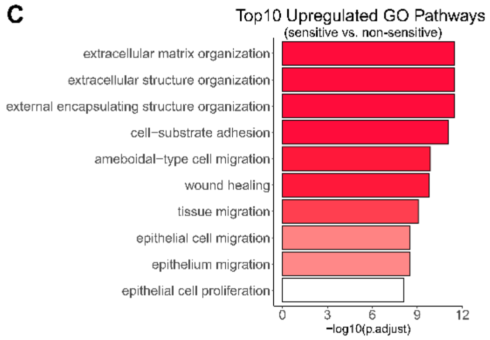

D

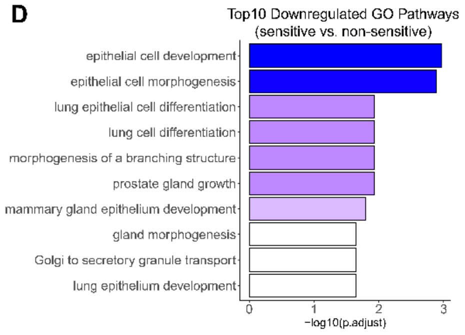

E

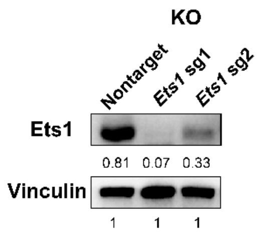

F

GDSC2 database

IC_{50}

BRCA subtype	Cell line NO.	Smallest average IC_{50} drugs
LA	7	15
LB	5	7
H	5	124
TNA	10	96
TNB	9	35
Total	36	275

P value

Dasatinib: Unique sensitive to basal B

G

Drug	Pathway	Target	Subtype IC_{50}(μM)					P value (vs BaB)			
			LA	LB	H	BaA	BaB	LA	LB	H	BaA
Bortezomib	Protein stability and degradation	Proteasome	-4.37	-3.63	-4.64	-5.19	-5.52	*	ns	ns	ns
Staurosporine	RTK signaling	Broad spectrum kinase inhibitor	-0.93	-1.98	-2.26	-2.91	-3.59	**	*	*	ns
Luminespib	Protein stability and degradation	HSP90	-1.01	-2.27	-2.73	-2.31	-2.81	*	ns	ns	ns
Dactolisib	PI3K/MTOR signaling	PI3K (class 1), MTORC1, MTORC2	-1.10	-0.94	-1.49	-1.40	-2.02	ns	ns	ns	ns
MG-132	Protein stability and degradation	Proteasome, CAPN1	-0.48	-0.06	-0.94	-1.42	-1.53	ns	ns	*	ns
Epirubicin	DNA replication	Anthracycline	0.62	1.18	-0.33	-0.62	-0.65	ns	ns	*	ns
LMP744	DNA replication	TOP1	2.44	4.64	-0.16	0.59	-0.27	*	***	ns	ns
AZD7762	Cell cycle	CHEK1, CHEK2	2.21	3.42	0.20	-0.20	-0.20	***	*	ns	ns
Dasatinib	Other, kinases	ABL, SRC, Ephrins, PDGFR, KIT	3.79	3.60	2.93	1.98	0.15	**	**	**	*
Telomerase Inhibitor IX	Genome integrity	Telomerase	1.00	2.16	0.99	0.48	0.46	ns	ns	***	ns
Teniposide	DNA replication	/	2.67	3.49	0.57	1.36	0.55	*	*	ns	ns
MK-1775	Cell cycle	WEE1, PLK1	2.76	3.08	1.84	0.83	0.64	***	*	ns	ns
Navitoclax	Apoptosis regulation	BCL2, BCL-XL, BCL-W	1.82	2.24	1.84	1.55	1.07	ns	ns	*	ns
Foretinib	RTK signaling	MET, KDR, TIE2, VEGFR3/FLT4, RON, PDGFR, FGFR1, EGFR	2.66	2.64	2.34	1.28	1.15	ns	*	ns	ns
TW 37	Apoptosis regulation	BCL2, BCL-XL, MCL1	2.42	3.09	1.79	1.37	1.25	/	/	/	/
123829	Unclassified	/	2.79	3.37	1.30	2.43	1.27	ns	*	ns	ns
Wee1 Inhibitor	Cell cycle	WEE1, CHEK1	2.59	3.57	2.13	1.78	1.52	*	*	ns	ns
IGF1R_3801	IGF1R signaling	IGFR1	3.89	5.05	2.93	1.85	1.68	*	*	ns	ns
Cediranib	RTK signaling	VEGFR, FLT1, FLT2, FLT3, FLT4, KIT, PDGFRB	3.01	3.50	3.00	2.47	1.89	ns	ns	ns	ns
BMS-536924	IGF1R signaling	IGF1R, IR	4.07	4.75	3.34	2.71	1.95	****	***	ns	ns
AZ960	Other, kinases	JAK2, JAK3	4.18	3.17	2.61	2.36	2.03	**	ns	ns	ns
BDP-00009066	Cytoskeleton	MRCKB_HUMAN	3.65	4.38	2.82	2.54	2.03	*	*	ns	ns
ERK_2440	ERK MAPK signaling	ERK1,ERK2	4.41	4.62	2.85	2.23	2.14	**	**	ns	ns
Mycophenolic acid	Unclassified	/	3.43	4.12	2.63	2.26	2.16	ns	ns	ns	ns
AZD4547	RTK signaling	FGFR1, FGFR2, FGFR3	2.86	3.58	3.48	2.48	2.18	ns	ns	ns	ns
UMI-77	Apoptosis regulation	MCL1	3.07	4.22	2.41	2.52	2.30	ns	ns	ns	ns
Nilotinib	ABL signaling	ABL	2.78	3.42	2.86	2.79	2.44	ns	ns	ns	ns
Bleomycin	DNA replication	dsDNA break induction	3.52	4.39	3.05	3.04	2.80	ns	*	ns	ns
NVP-ADW742	IGF1R signaling	IGF1R	4.51	4.07	3.79	3.22	3.13	ns	ns	*	ns
AZD1332	RTK signaling	NTRK1, NTRK2, NTRK3	4.83	5.61	3.66	4.37	3.50	ns	ns	ns	ns
Fludarabine	DNA replication	Antimetabolite	5.64	5.51	5.11	4.84	3.69	ns	ns	ns	ns
PD173074	RTK signaling	FGFR1, FGFR2, FGFR3	5.22	5.15	4.00	4.49	3.71	*	*	ns	ns
PFI-1	Chromatin other	BRD4	4.83	5.06	4.82	4.16	4.11	ns	ns	ns	ns
Avagacestat	Other	Amyloid beta20, Amyloid beta40	5.98	5.58	4.47	4.64	4.46	*	*	ns	ns

Supplementary Figure S2. Dasatinib exhibits high sensitivity in basal B subtypes with high *ETS1* activity.

(**A**)The scatter plot represents the IC_{50} values across 36 breast cancer cell lines from the GDSC2 database treated with cisplatin, bosutinib, paclitaxel, and docetaxel in different subtypes, and each dot represents the IC_{50} value for one cell line.

(**B**) Kinase inhibition profiling compounds at 0.5 μM in the presence of 10 μM ATP, based on published data. Residual kinase activity was quantified for each kinase–substrate pair.

(**C**) Top 10 upregulated Gene Ontology (GO) pathways, ranked by Benjamini–Hochberg adjusted p-value.

(**D**) Top 10 downregulated GO pathways, ranked by Benjamini–Hochberg adjusted p-value.

(**E**) Western blot confirming knockout (KO) of *Ets1* in 4T1 cells. Quantified data were normalized to the reference control of vinculin.

(**F**) Schematic illustration of data analysis from the GDSC2 dataset.

(**G**) The information of 35 drugs for which the lowest average IC_{50} subtype corresponds to the basal B subtype.
