# Supplementary figures and images for: Dasatinib Inhibits Basal B Breast Cancer Through ETS1-Mediated Extracellular Matrix Remodeling
Source: Biomedicines. 2025 Nov 26;13(12):2888. doi: 10.3390/biomedicines13122888 (PMC12730708; doi:10.3390/biomedicines13122888)

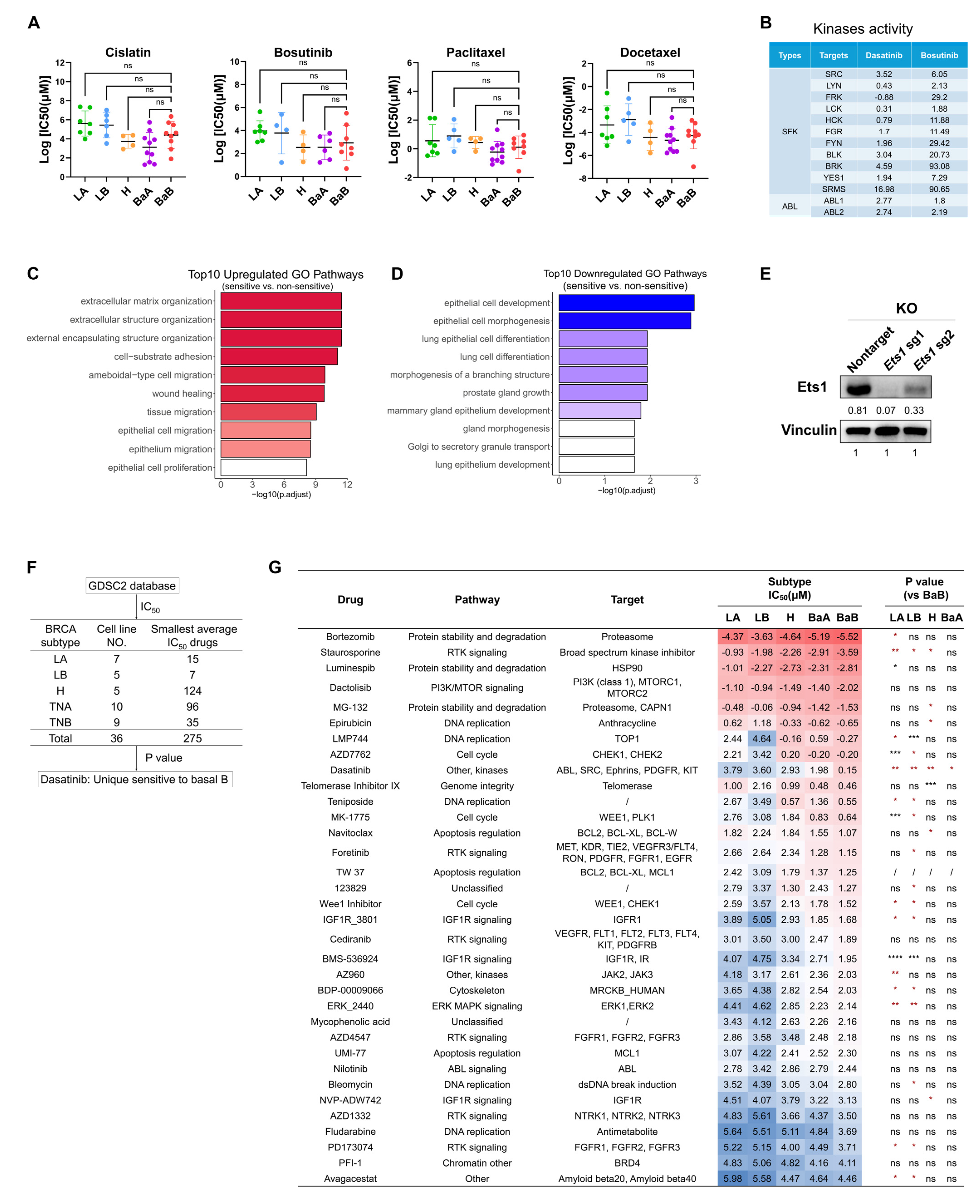

Supplement: Supplementary file 1 [file biomedicines-13-02888-s001.zip › Supplementary Figure S2.tif]
